# Supplementary material for: Starvation in Mice Induces Liver Damage Associated with Autophagy
Source: Nutrients. 2024 Apr 17;16(8):1191. doi: 10.3390/nu16081191 (PMC11053507; doi:10.3390/nu16081191)
Supplement: Supplementary file 1 [file nutrients-16-01191-s001.zip › nutrients-2919872-supplementary.pdf]

Reference	Year	Number of patients	Sex	Body Mass Index (BMI) (kg/m²) ± SD	Age ± SD	Findings
Mickley et al. [1]	1996	879	F and M (13)	20 ± 2.9	22.51 ± 7	Elevation of transaminases in 4% of anorectic (as well as anorectic and bulimic) patients.
Miller et al. [2]	2005	214	F	16.8 ± 1.4	25 ± 6.4	Elevation of ALT in 12% of patients.
Montagnese et al. [3]	2007	97	F	15.6 ± 1.4	23.3 ± 5.5	Elevation of transaminases in 20% of patients with BMI ≤ 16 kg/m².
Fong et al. [4]	2008	53	F	18 ± 1.6	18.5 ± 2.9	Elevation of ALT in 26% and AST in 19% of patients.
Rautou et al. [5]	2008	12	F and M (2)	11.3 ± 1.1	25.7 ± 7.7	Examination of liver biopsies from patients. The study neither found signs of hepatocyte necrosis nor apoptosis, instead autophagosomes and glycogen depletion were observed. Transaminases elevation in all patients.
Tsukamoto et al. [6]	2008	25	F	15.2 ± NR	27 ± 9	Elevation of transaminases in 52% of patients.
Gaudiani et al. [7]	2012	25	F and M (3)	13.1 ± 2.1	26 ± 7	Mild elevation of transaminases (up to three times of the normal values) in 32% of patients, severe elevation (values greater than three times of the normal) in 44% of patients.
Hanachi et al. [8]	2013	126	F and M ()	12 ± 1.8	30 ± 10.8	Elevation of transaminases in 43% of patients.
Nagata et al. [9]	2015	356	F and M (39)	15.9 ± 1.9	16.1 ± 2.4	Elevation of ALT was observed in 41.1% of patients. Male sex and low BMI led to increased odds of ALT elevations, while age and duration of illness did not affect odds.
Rosen et al. [10]	2016	181	F and M (19)	12.8 ± 1.8	27 ± 5	Mild elevation of transaminases (up to three times of the normal values) in 27% of patients, severe elevation (values greater than three times than normal) in 35% of patients.
Fanin et al. [11]	2020	34	F	15.6 ± 2.1	25 ± 6.3	Elevation of ALT in 29% of patients. Ultrasonography indicated mild steatosis in 47% of patients.
Cuntz et Vorderholzer [12]	2022	3755	F and M (157)	11.5 ± NR	31.5 ± NR	Elevation of transaminases in >50% of patients.

Case studies reporting starvation-induced liver damage in AN						
**Reference**	**Year**	**Number of patients**	**Sex**	**Body Mass Index (BMI) (kg/m²)**	**Age**	**Findings**
Yaryura-Tobias et al. [13]	2000	1	F	14.2	23	Patient with diabetic ketoacidosis. Signs of hepatic steatosis could be observed in computed tomography scans.
De Caprio et al. [14]	2006	2	F	14.1	18	Patient with acute liver failure and signs of moderate steatosis in sonography.
				13.2	30	Patient with signs of acute liver failure, normalizing after weight gain.
Sakada et al. [15]	2006	1	F	7.6	20	Patient presenting with signs of mild hepatic steatosis and liver atrophy in CT scans. Transaminase elevations were observed. While the patient's nutritional condition improved, the patient developed a fatal hepatic failure. Autopsy findings confirmed the presence of hepatic steatosis and reported pericellular fibrosis.
Harris et al. [16]	2012	1	F	10.7	34	Patient with severe transaminase elevation. A liver biopsy showed mild cell swelling, mild fibrosis and mild macro-vesicular steatosis.
Restellini et al. [17]	2013	1	F	14	24	Patient with elevated transaminases, liver biopsy with signs of autophagy.
Ramsoekh et al. [18]	2014	1	F	12.4	43	Patient with elevated transaminases. Liver biopsy with several apoptotic hepatocytes and iron deposition.
Takata et al. [19]	2021	1	F	14	36	Patient with ascites, gastroesophageal varices and elevation of transaminase values. Liver biopsy with mild steatosis, mild chronic inflammation, fibrosis and benign nodular lesions.
Su et al. [20]	2021	1	F	14.2	45	Patient with elevated transaminases. Liver biopsy showed mild depletion of glycogen and scattered atrophy of hepatocytes.
Faragalla et al. [21]	2022	1	F	12.7	19	Patient with elevation of transaminase values. Liver biopsy with glycogen depletion, iron deposition and hyperplastic Kupffer cells.
Sakata et al. [22]	2022	1	F	11.2	34	Patient with hypoglycemic coma and elevated transaminase values.
Wallace et al. [23]	2023	1	F	10.3	30	Patient with hypoglycemia, elevation of transaminases, which decreased when enteral feeding was initiated.

References

1. Mickley, D., et al., *Abnormal liver enzymes in outpatients with eating disorders.* Int J Eat Disord, 1996. 20(3): p. 325-9.
2. Miller, K.K., et al., *Medical findings in outpatients with anorexia nervosa.* Arch Intern Med, 2005. 165(5): p. 561-6.
3. Montagnese, C., et al., *Cholinesterase and other serum liver enzymes in underweight outpatients with eating disorders.* Int J Eat Disord, 2007. 40(8): p. 746-50.
4. Fong, H.F., et al., *Prevalence and predictors of abnormal liver enzymes in young women with anorexia nervosa.* J Pediatr, 2008. 153(2): p. 247-53.
5. Rautou, P.E., et al., *Acute liver cell damage in patients with anorexia nervosa: a possible role of starvation-induced hepatocyte autophagy.* Gastroenterology, 2008. 135(3): p. 840-8, 848 e1-3.
6. Tsukamoto, M., et al., *Hepatocellular injuries observed in patients with an eating disorder prior to nutritional treatment.* Intern Med, 2008. 47(16): p. 1447-50.
7. Gaudiani, J.L., et al., *Severe anorexia nervosa: outcomes from a medical stabilization unit.* Int J Eat Disord, 2012. 45(1): p. 85-92.
8. Hanachi, M., J.C. Melchior, and P. Crenn, *Hypertransaminasemia in severely malnourished adult anorexia nervosa patients: risk factors and evolution under enteral nutrition.* Clin Nutr, 2013. 32(3): p. 391-5.
9. Nagata, J.M., et al., *Associations of elevated liver enzymes among hospitalized adolescents with anorexia nervosa.* J Pediatr, 2015. 166(2): p. 439-43 e1.
10. Rosen, E., et al., *Liver dysfunction in patients with severe anorexia nervosa.* Int J Eat Disord, 2016. 49(2): p. 151-8.
11. Fanin, A., et al., *Liver alterations in anorexia nervosa are not caused by insulin resistance.* Intern Emerg Med, 2020. 15(2): p. 337-339.
12. Cuntz, U. and U. Voderholzer, *Liver Damage Is Related to the Degree of Being Underweight in Anorexia Nervosa and Improves Rapidly with Weight Gain.* Nutrients, 2022. 14(12).
13. Yaryura-Tobias, J.A., A. Pinto, and F. Neziroglu, *Anorexia nervosa, diabetes mellitus, brain atrophy, and fatty liver.* Int J Eat Disord, 2001. 30(3): p. 350-3.
14. De Caprio, C., et al., *Severe acute liver damage in anorexia nervosa: two case reports.* Nutrition, 2006. 22(5): p. 572-5.
15. Sakada, M., et al., *Severe steatosis resulted from anorexia nervosa leading to fatal hepatic failure.* J Gastroenterol, 2006. 41(7): p. 714-5.
16. Harris, R.H., G. Sasson, and P.S. Mehler, *Elevation of liver function tests in severe anorexia nervosa.* Int J Eat Disord, 2013. 46(4): p. 369-74.
17. Restellini, S., L. Spahr, and L. Rubbia Brandt, *Severe starvation-induced hepatocyte autophagy as a cause of acute liver injury in anorexia nervosa: a case report.* Case Reports Hepatol, 2013. 2013: p. 749169.
18. Ramsoekh, D., P. Taimr, and T. Vanwolleghem, *Reversible severe hepatitis in anorexia nervosa: a case report and overview.* Eur J Gastroenterol Hepatol, 2014. 26(4): p. 473-7.
19. Takata, K., et al., *Gastroesophageal Varices and Hyperplastic Nodules of the Liver in a Patient with Anorexia Nervosa.* Intern Med, 2021. 60(19): p. 3107-3112.
20. Su, A., et al., *Two Acute Liver Injuries in a Patient With Malnutrition.* J Med Cases, 2021. 12(8): p. 315-318.
21. Faragalla, K., et al., *Value of liver biopsy in anorexia nervosa-related transaminitis: A case study and literature review.* Hepatol Res, 2022. 52(7): p. 652-658.
22. Sakata, M., et al., *Pathogenesis of Severe Liver Injury in Patients with Anorexia Nervosa: A Report of Two Cases and a Literature Review.* Kurume Med J, 2022. 67(2.3): p. 121-129.
23. Wallace, J.M., et al., *A Hepatic Manifestation of Anorexia Nervosa.* Eur J Case Rep Intern Med, 2023. 10(5): p. 003675.
